# Comparative study of machine learning techniques for post-combustion carbon capture systems

**DOI:** 10.3389/frai.2024.1441934

**Published:** 2024-11-14

**Authors:** Yeping Hu, Bo Lei, Yash Girish Shah, Jose Cadena, Amar Saini, Grigorios Panagakos, Phan Nguyen

**Affiliations:** ^1^Lawrence Livermore National Laboratory, Livermore, CA, United States; ^2^National Energy Technology Laboratory, Pittsburgh, PA, United States; ^3^NETL Support Contractor, Pittsburgh, PA, United States; ^4^Department of Chemical Engineering, Carnegie Mellon University, Pittsburgh, PA, United States

**Keywords:** machine learning, graph neural networks, convolutional neural networks, computational fluid dynamics, carbon capture systems

## Abstract

Computational analysis of countercurrent flows in packed absorption columns, often used in solvent-based post-combustion carbon capture systems (CCSs), is challenging. Typically, computational fluid dynamics (CFD) approaches are used to simulate the interactions between a solvent, gas, and column's packing geometry while accounting for the thermodynamics, kinetics, heat, and mass transfer effects of the absorption process. These simulations can then be used explain a column's hydrodynamic characteristics and evaluate its CO_2_-capture efficiency. However, these approaches are computationally expensive, making it difficult to evaluate numerous designs and operating conditions to improve efficiency at industrial scales. In this work, we comprehensively explore the application of statistical ML methods, convolutional neural networks (CNNs), and graph neural networks (GNNs) to aid and accelerate the scale-up and design optimization of solvent-based post-combustion CCSs. We apply these methods to CFD datasets of countercurrent flows in absorption columns with structured packings characterized by several geometric parameters. We train models to use these parameters, inlet velocity conditions, and other model-specific representations of the column to estimate key determinants of CO_2_-capture efficiency without having to simulate additional CFD datasets. We also evaluate the impact of different input types on the accuracy and generalizability of each model. We discuss the strengths and limitations of each approach to further elucidate the role of CNNs, GNNs, and other machine learning approaches for CO_2_-capture property prediction and design optimization.

## 1 Introduction

**Figure 1:**
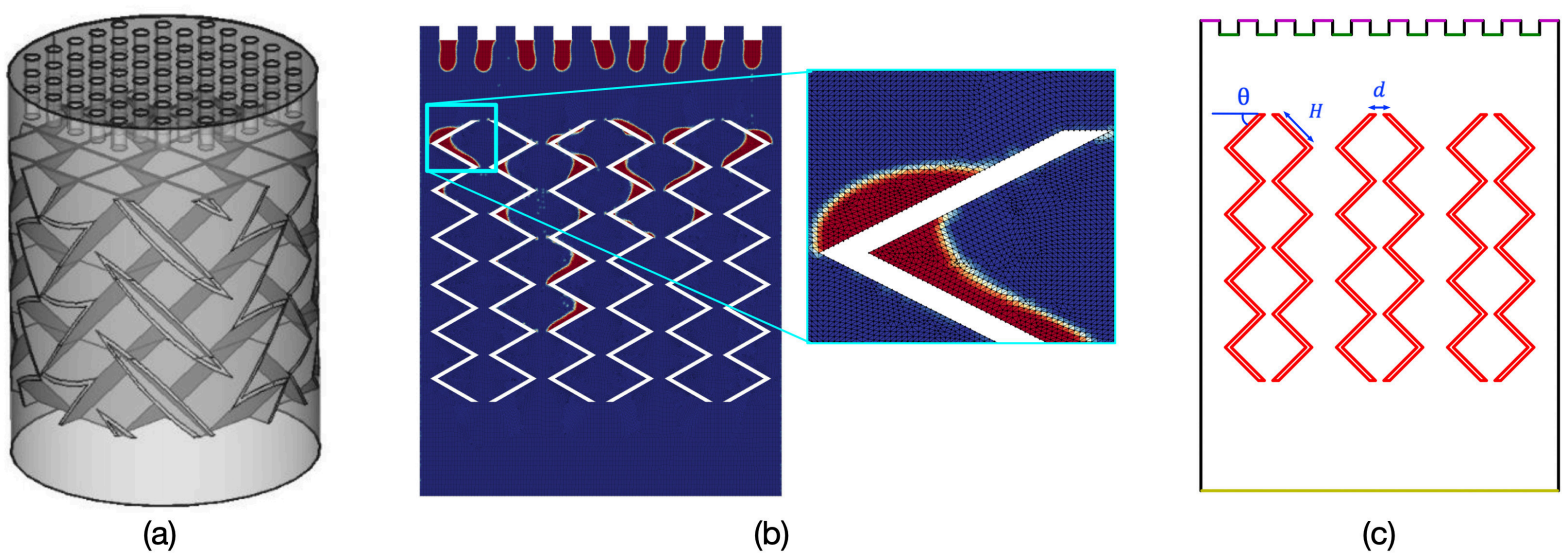
**(a)** Illustration of 3D CCS with packings placed inside the reactor column; **(b)** CFD simulation of a 2D slice at a given time step and a zoomed view of triangular meshes; **(c)** structured packing geometry (defined with three parameters) and colored node types: column walls, packing, inlet, gas outlet, pressure outlet.

Electricity generation is a main contributor to global greenhouse gas emissions, and reducing the carbon intensity of this process is critical to reducing greenhouse gas concentrations to safe, sustainable levels (Arto and Dietzenbacher, 2014). Mitigating emissions from fossil-based power plants can be achieved with various CO_2_-capture technologies. Typically, CO_2_-capture from flue gas is accomplished using pre-combustion, oxyfuel-combustion, or post-combustion technologies (Koytsoumpa et al., 2018). Among these, the most utilized approach is solvent-based post-combustion, wherein CO_2_ is absorbed through interactions between a liquid solvent and flue gas inside a reactor column filled with packings (Wang et al., 2017); an example of a column is shown in Figure 1a. Packings are structured materials placed inside the column to help distribute the flow of a solvent throughout the column and increase the contact surface between the solvent and flue gas, thereby enhancing the efficiency of CO_2_ absorption. A fundamental challenge in designing such solvent-based carbon capture systems (CCSs) is optimizing the selection of solvent, packing geometry, and operating conditions to maximize CO_2_-capture. The combination of the large design space of parameters and complex interactions between the solvent, gas, and packings makes it difficult to find an optimal configuration that maximizes CO_2_-capture efficiency while minimizing costs associated with searching, building, and testing a candidate design of a CCS.

Computational fluid dynamics (CFD) simulations can provide detailed insights into the fluid flow and interactions between solvents and CO_2_-rich flue gases without physical testing, making it possible to virtually evaluate a wide range of alternative designs quickly and cost-effectively. These simulations are crucial for reducing product-development cycles and scaling up from lab to industrial applications, ensuring robust and scalable designs (Mudhasakul et al., 2013; Razavi et al., 2013). However, generating CFD simulations is computationally expensive, presenting a major bottleneck in evaluating potential configurations within a CCS design optimization process. As a result, there has been a growing interest in using machine learning (ML) to accelerate CFD simulations and obtain near-real-time predictions (Bhatnagar et al., 2019; Kochkov et al., 2021; Thuerey et al., 2020). Carbon capture technologies have seen emerging applications of ML to both large-scale (industries) and small-scale (R&D and laboratory-scale) problems, including optimizing flow operating conditions and screening ionic liquids, adsorbents, and membranes (Shalaby et al., 2021; Venkatraman and Alsberg, 2017; Meng et al., 2019; Zhang et al., 2022). Additional backgound information and related works can be found in Supplementary material, Section 3.

In this study, we evaluate the performance of statistical ML methods, convolutional neural networks (CNNs), and graph neural networks (GNNs) in predicting CO_2_-capture efficiency metrics. Our results indicate that the GNN-based model outperforms other methods in terms of prediction accuracy. We also highlight the importance of using detailed data representations, such as images and structured graphs, to enhance predictive performance. The findings of this research provide valuable guidance for selecting appropriate ML algorithms and demonstrate the potential of leveraging these models to identify optimal packing geometries and operating conditions.

## 2 Method

### 2.1 Dataset description

We focus on a CO_2_-capture column with periodic or structured packings, which is commonly used in solvent-based post-combustion CCSs. Within these columns, CO_2_ is captured through an absorption process caused by the interaction between a liquid solvent and a CO_2_-laden gas. The structured packings help distribute the flow of solvent and increase the surface area of interaction. Figure 1b shows a CFD simulation snapshot of countercurrent flow occurring in a 2D bench-scale column, in which solvent is injected into the column from the top “inlets” and a compressible ideal gas is injected below.

Two key CO_2_-capture efficiency metrics that can be derived from CFD simulations of this flow are the *interfacial area* and the *wetted area*. The interfacial area represents the total surface area where the liquid solvent meets the CO_2_-laden gas. The rate of CO_2_ absorption is correlated with the available interfacial area for mass transfer between the gas and liquid phases; an increased interfacial area provides more points of interaction, which can enhance the CO_2_ absorption efficiency (Ataki and Bart, 2006; Tsai et al., 2011). The wetted area measures the surface area of the packing materials that is in contact with the liquid solvent (Bolton et al., 2019). Proper wetting of the packing materials is crucial for ensuring optimal effective mass transfer between the two phases, and the amount of wetted area can influence how effectively the solvent is distributed and retained, thereby affecting its contact with the gas (Singh et al., 2022).

Due to the high computational costs of generating a CFD simulation and the labor, material, and other expenses required to physically build a column, we seek to train ML models to accurately estimate CO_2_-capture efficiency metrics based on the physical geometry of a column and its expected operating conditions to screen designs, without having to compute additional CFD simulations or physically build a candidate design. However, to train ML models, we still require an initial dataset of CFD simulations of countercurrent flows from which we can compute these metrics. We use Ansys Fluent (Ansys, 2011) to model the fluid dynamics and chemical interactions within a CO_2_-capture column under various operating conditions and packing geometry configurations. In particular, we consider two inlet velocity values, 0.01 and 0.05 m/s, which describe the flow rate of the solvent into the column. For small inlet velocities, the wetted area may be more stable and predictable, but the interfacial area may be less effective due to lower turbulence (Zhu et al., 2020). In contrast, for large inlet velocities, the wetted area may be more variable due to increased turbulence, which may enhance the interfacial area by improving the contact between the phases, thus potentially increasing the efficiency of CO_2_ capture (Zhu et al., 2020). We also consider structured packing geometries that are parameterized by three structural variables: θ, *H*, and *d*. Figure 1c shows a representative column; θ describes the angle of a packing unit, *H* describes the length of unit, and *d* describes the distance between units. We consider the following values: θ∈[30, 45, 65], *H*∈[10, 13, 14.8], and *d*∈[1.78, 2.68, 3.56].

For each combination of inlet velocity and packing configuration, we generate a simulation in Ansys Fluent using a detailed two-phase reacting flow model (see Prosperetti and Tryggvason, 2009; Brackbill et al., 1992; Panagakos and Shah, 2023 for further details). We use three types of data representations in our machine learning models: a 3-parameter representation (θ, *H, d*), an image-based representation of the CO_2_-capture column, and a graph-based representation derived from the CFD mesh used to generate the simulations in Ansys Fluent. To assess the impact of turbulent data, we consider three dataset variants based on the inlet velocity: one using data with an inlet velocity of 0.01 m/s only, another with 0.05 m/s only, and a third combining data from both inlet velocities. We divide each dataset into train and test splits using Latin hypercube sampling (Loh, 1996) to ensure sufficient coverage of the 3-parameter space in the training set. In the combined velocity dataset, we include the inlet velocity value as an additional input modeling feature. These scenarios allow us to better assess each model's performance under specific conditions (single velocity) and its ability to make predictions across varied operational conditions (combined velocities). If our ML models are trained accurately with these simulations, additional CFD simulations will not be required to estimate the efficiency measures of other unseen parameter and operating configurations. Additional data processing details can be found in Supplementary material, Section 1.1.

### 2.2 Methods details

#### 2.2.1 Statistical ML methods

We first consider a set of baseline statistical ML methods, including *elastic net, lasso regression, linear regression, partial least squares regression*, and *ridge regression*, to predict CO_2_-capture efficiency metrics. To apply these methods, we use the 3-parameter representation of the packing geometries and the inlet velocity as inputs. Some of the major advantages of this approach include the simplicity of the data representation, availability and ease of use of the methods, computational efficiency, potential robustness to overfitting with proper training, and model interpretability. Because of the simplicity of the methods and the input data representation, this approach serves as a benchmark for the more complex data representations and advanced ML algorithms that we will consider. We implemented these methods using scikit-learn and optimized their hyperparameters through cross-validation.

The data representation used in this approach, however, may be too simplistic and limited for practical application and extrapolation. While the 3-parameter model efficiently encapsulates the basic dimensions and arrangements of the design shown in Figure 1c, it cannot fully account for more complex or novel packing geometries. First, the model will be strongly limited to the domain covered by the training dataset's parameter space. In addition, the model cannot be used to make predictions about CO_2_-capture efficiency when the design of the column is scaled or augmented, such as by expanding the column's width or height, and increasing or lengthening the pairs of packings. More advanced ML architectures can potentially overcome these limitations; methods such as CNNs and GNNs are capable of capturing spatial hierarchies and complex topologies within data, thus providing a more detailed understanding of fluid dynamics and interactions within varied packing geometries.

Overall, while statistical ML methods are computationally efficient and provide a solid foundation for initial modeling, their inability to fully extrapolate to more complex geometries limits their potential in scaling and optimizing CCS designs. More sophisticated techniques may yield significant improvements in predictive accuracy and design flexibility. To this end, we consider CNNs and GNNs.

#### 2.2.2 Convolutional neural networks

Since the computational mesh used to generate CFD simulations of the CO_2_-capture column can also be represented as an image by using a grid interpolation, we next consider applications of CNNs to predict CO_2_-capture efficiency. CNNs are a class of deep learning models widely used for image classification, object detection, and other visual recognition tasks (Li et al., 2021) and have also been used in other CO_2_-capture-related applications (Zhou et al., 2019; Kaur et al., 2023). Here, we adapt the LeNet architecture (LeCun et al., 1998) and hypothesize that the CNN can use the image representation of the column and analyze the spatial complexities of the walls, packing structures, inlets, outlets, and other physical components to make accurate predictions of the interfacial area and wetted area.

We consider two types of image representations of the column. First, we interpolate the CFD mesh onto a 3 × 128 × 128 colored image. A colored image allows us to distinctly represent the various physical components of the column, each differentiated by unique colors (see Figure 1c). Compared to the 3-parameter representation used in the statistical ML models, these images provide richer detail about the spatial and structural components of the column and provide an avenue to extrapolate to structures that cannot be represented by the three parameters. However, CNNs typically require more computational power and, given the size of our dataset, may be prone to overfitting. We therefore also interpolate the mesh onto a 1 × 128 × 128 grayscale image, which simplifies the representation by translating all features into shades of black and white but eliminates the explicit distinction between different types of boundaries and structures. The reduced dimensionality provides a more generalized view but forces a tradeoff between computational efficiency and data intricacy that may be critical for making accurate predictions about CO_2_-capture efficiency.

The LeNet architecture that we adapted to our use-case consists of two convolutional layers, each with a kernel size of 32, followed by three multi-layer perceptron (MLP) layers. We use ReLU activation functions throughout the model. To incorporate inlet velocity as an input, we use an additional linear layer to generate a 128-dimensional velocity embedding. This embedding is then concatenated with the LeNet model's output after the first two MLP layers. We then process the resulting vector through the third MLP layer to produce the final output prediction. We train the model using an Adam optimizer with a learning rate of 1e-4, 1,000 training steps, and a batch size of 8. Additional information on data processing and model details can be found in Supplementary material, Sections 1.2, 2.1.

CNNs provide a powerful tool for capturing and analyzing the intricate details of the packed column, allowing us to begin generalizing predictions to packing geometries that cannot be represented by the three-parameter representation. However, these models demand careful management of computational resources and model complexity. Furthermore, the models require a fixed image size as input, which prevents the models from generalizing to columns of different scales. For example, a column that has twice the physical height or width of the columns that our CNN is trained on still needs to be projected onto a 128 × 128 image to be processed by the CNN, but information about the details and scale of the column is lost. To address this weakness, we now consider GNNs.

#### 2.2.3 Graph neural networks

GNNs (Zhou et al., 2020) are a class of deep learning models designed to operate on graph-structured data, allowing them to capture relational information between entities. GNNs have demonstrated promising results in various application areas, including chemical reaction and property prediction (Do et al., 2019; Gilmer et al., 2017; Xie and Grossman, 2018; Sanyal et al., 2018; Nguyen et al., 2021; Zhang et al., 2024), fluid dynamics prediction (Hu et al., 2023), and CO_2_-capture-related applications (Jian et al., 2022; Bartoldson et al., 2023). Unlike CNNs, GNNs can operate on unstructured grids of arbitrary sizes and thus have better generalizability across various packing columns and scales. Since the computational mesh used to generate CFD simulations of the CO_2_-capture column is also a graph, we can apply GNNs to handle the complex geometries of the column. GNNs come in various architectures, each with unique approaches to aggregating and processing information across graph structures. Here we consider three commonly used models: Graph Convolutional Networks (GCN) (Zhang et al., 2019), Graph Attention Networks (GAT) (Veličković et al., 2017), and Graph Isomorphism Networks (GIN) (Xu et al., 2018).

We construct a graph by starting with the mesh representation of the packed columns used in the CFD simulations. Nodes in the graph represent different locations in the column. To incorporate geometric information, we include each node's position and one-hot encoded type as node features, and we include relative positions and distances between nodes as edge features. This approach allows our GNN-based models to effectively capture the spatial relationships and geometric characteristics of the column's packing geometry, scale to arbitrary mesh sizes, and infer on novel column designs.

On average, the graph of each column configuration consists of 183,844 nodes and 1,090,258 edges. To preprocess the mesh data (see Figure 1b) for GNN, we encode node and edge features using a linear layer to obtain 64-dimensional latent embedding vectors, which are then passed through eight GNN layers to perform message-passing and obtain node embeddings. We obtain the final graph embedding by applying max pooling to the node embeddings. Our experiments indicate that only considering nodes related to packing geometries during the pooling process yields better performance compared to pooling over all nodes. To incorporate inlet velocity as an input, we use an additional linear layer to generate a 64-dimensional velocity embedding. We then concatenate the graph and velocity embeddings and feed them through a two-layer MLP to obtain an output prediction. To train the model, we use a batch size of 4 and an Adam optimizer with a learning rate decayed from 1e-3 to 1e-5 over 1,000 training steps. Additional information on data processing and model details can be found in Supplementary material, Sections 1.3, 2.2.

Compared to the statistical ML- and CNN-based approaches, the GNN-based approach is more flexible in adapting to different packing geometries and scales. Since GNNs can process graphs of arbitrary sizes, we can infer CO_2_-capture efficiency measures for columns whose sizes are different from those of the training set by simply representing the new columns with a larger graph. In addition, we can perform inference for packing geometries not captured by the 3-parameter representation (Figure 1c) by assigning the node types correctly in the new geometries. Therefore, GNNs can provide more flexibility in evaluating a broader range of designs and operational scenarios within a design optimization pipeline.

## 3 Results

**Figure 2:**
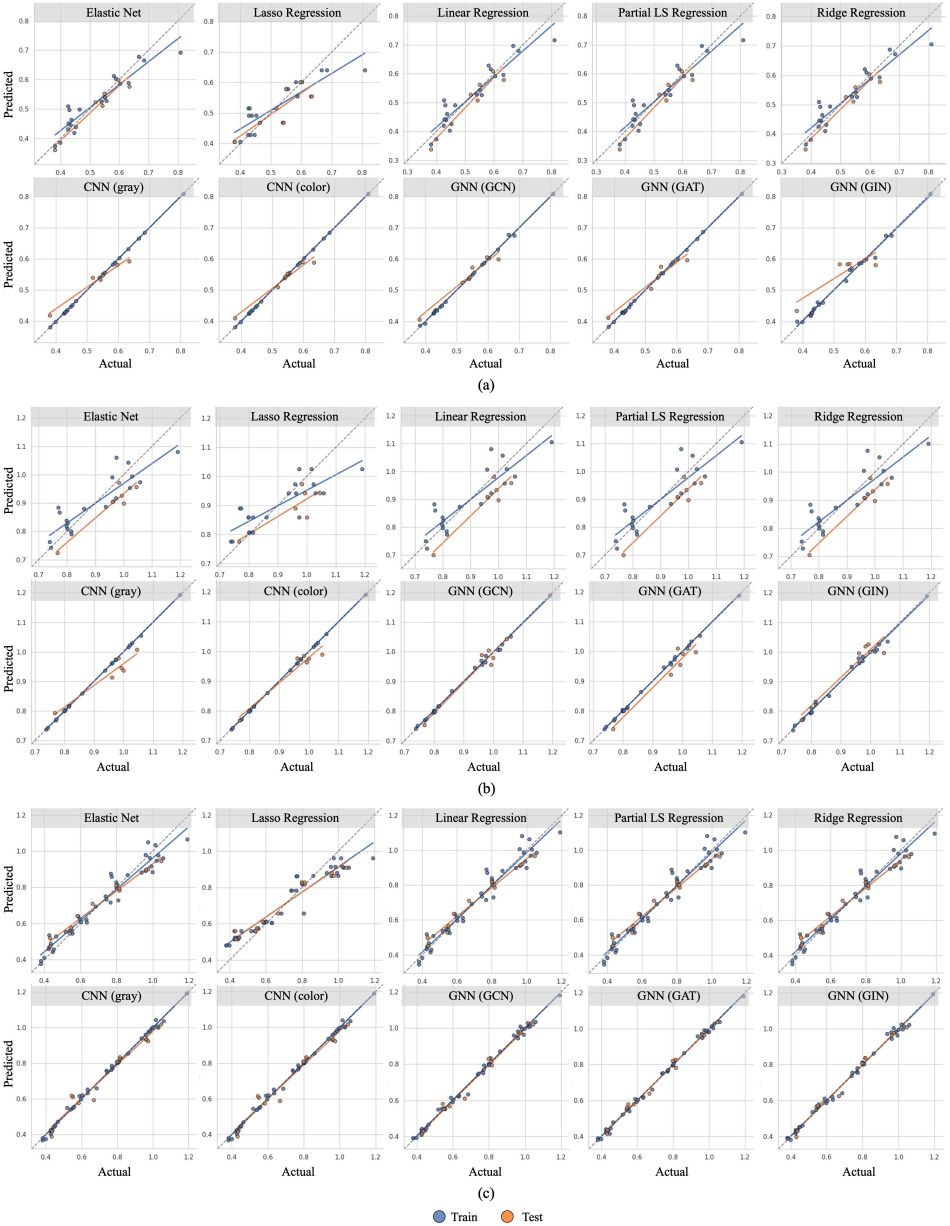
Relationship between predicted (mean value across all seeds) and actual interfacial area (IA) values for various models and various inlet velocities (i.e. 0.01 m/s, 0.05 m/s, and combined). Blue dots represent training data, while orange dots represent test data. The blue and orange lines are linear regression lines for blue and orange dots, respectively. The black dotted line indicates perfect prediction. Dots closer to this line and the alignment of the regression lines with the dotted line reflect better model accuracy and generalization. **(a)** Inlet velocity = 0.01 m/s. **(b)** Inlet velocity = 0.05 m/s. **(c)** Inlet velocity = 0.01 m/s and 0.05 m/s.

**Figure 3:**
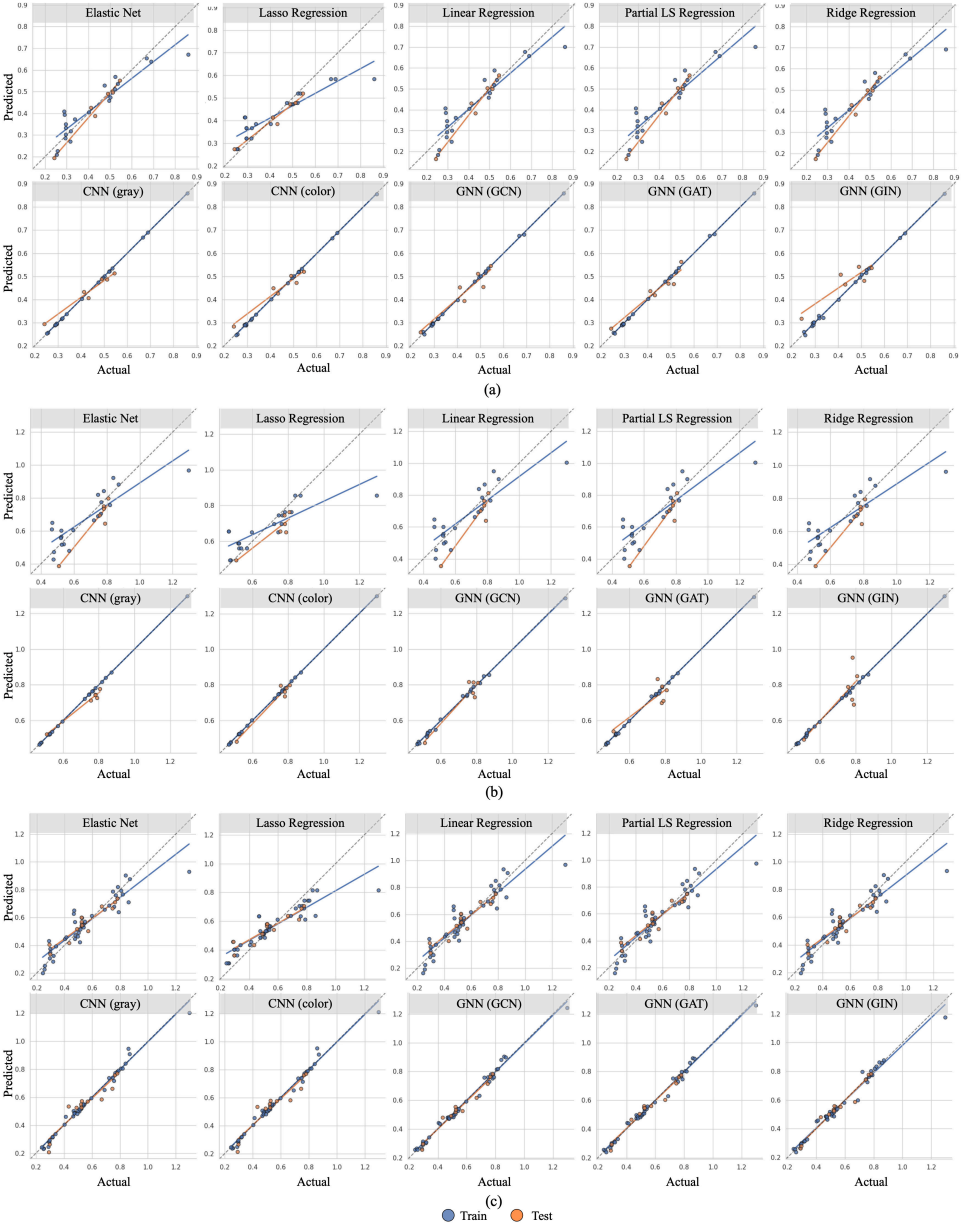
Relationship between predicted (mean value across all seeds) and actual wetted area (WA) values for various models and various inlet velocities (i.e. 0.01 m/s, 0.05 m/s, and combined). Blue dots represent training data, while orange dots represent test data. The blue and orange lines are linear regression lines for blue and orange dots, respectively. The black dotted line indicates perfect prediction. Dots closer to this line and the alignment of the regression lines with the dotted line reflect better model accuracy and generalization. **(a)** Inlet velocity = 0.01 m/s. **(b)** Inlet velocity = 0.05 m/s. **(c)** Inlet velocity = 0.01 m/s and 0.05 m/s.

We evaluate the accuracy of our models using three metrics: *R-squared* (*R*^2^), *Root Mean Square Error (RMSE)*, and *Mean Absolute Percentage Error (MAPE)*. Comparisons of these metrics and predictions for our statistical ML, CNN-based, and GNN-based models are shown in Table 1 and Figures 2, 3.

**Table 1:** A comparative analysis of various ML models in their ability to predict CO_2_-capture efficiency metrics.

	**Interfacial area**			**Wetted area**		
	*R*^2^↑	**RMSE** ↓	**MAPE** ↓	*R*^2^↑	**RMSE** ↓	**MAPE** ↓
**Inlet velocity @ 0.01 m/s**					
Elastic net	0.876	0.028	**0.037**	**0.919**	**0.028**	**0.064**
Lasso regression	0.649	0.047	0.066	0.913	0.029	0.065
Linear regression	0.833	0.033	0.054	0.836	0.040	0.096
Partial least squares	0.833	0.033	0.054	0.836	0.040	0.096
Ridge regression	0.854	0.030	0.046	0.868	0.036	0.086
CNN (gray)	**0.899** **±** **0.036**	**0.025** **±** **0.004**	0.041 ± 0.007	0.875 ± 0.029	0.035 ± 0.004	0.079 ± 0.012
CNN (color)	0.872 ± 0.119	0.027 ± 0.013	0.038 ± 0.019	0.844 ± 0.047	0.039 ± 0.006	0.082 ± 0.026
GNN (gcn)	0.814 ± 0.076	0.034 ± 0.008	0.057 ± 0.015	0.855 ± 0.039	0.037 ± 0.005	0.083 ± 0.017
GNN (gat)	0.711 ± 0.193	0.040 ± 0.015	0.063 ± 0.025	0.834 ± 0.119	0.038 ± 0.013	0.081 ± 0.027
GNN (gin)	0.667 ± 0.182	0.045 ± 0.011	0.074 ± 0.020	0.730 ± 0.156	0.049 ± 0.014	0.105 ± 0.032
**Inlet velocity @ 0.05 m/s**					
Elastic net	0.417	0.068	0.063	0.337	0.084	0.102
Lasso regression	0.194	0.080	0.065	0.389	0.081	0.090
Linear regression	0.403	0.069	0.064	0.199	0.092	0.108
Partial least squares	0.403	0.069	0.064	0.199	0.092	0.108
Ridge regression	0.408	0.069	0.064	0.355	0.083	0.101
CNN (gray)	0.764 ± 0.097	0.043 ± 0.010	0.036 ± 0.006	0.832 ± 0.099	0.041 ± 0.013	0.049 ± 0.019
CNN (color)	0.844 ± 0.056	0.035 ± 0.007	0.027 ± 0.006	**0.855** **±** **0.064**	0.038 ± 0.010	**0.041** **±** **0.005**
GNN (gcn)	0.782 ± 0.159	0.039 ± 0.014	0.034 ± 0.011	0.848 ± 0.127	**0.037** **±** **0.016**	0.043 ± 0.016
GNN (gat)	0.752 ± 0.076	0.044 ± 0.007	0.039 ± 0.004	0.676 ± 0.059	0.059 ± 0.005	0.068 ± 0.003
GNN (gin)	**0.869** **±** **0.065**	**0.031** **±** **0.007**	**0.026** **±** **0.005**	0.620 ± 0.244	0.060 ± 0.022	0.064 ± 0.020
**Inlet velocity @ 0.01 & 0.05 m/s**					
Elastic net	0.926	0.056	0.071	0.868	0.058	0.105
Lasso regression	0.873	0.073	0.085	0.762	0.078	0.136
Linear regression	0.954	0.044	0.056	0.888	0.053	0.096
Partial least squares	0.954	0.044	0.057	0.886	0.054	0.095
Ridge regression	0.948	0.047	0.060	0.868	0.058	0.105
CNN (gray)	0.956 ± 0.001	0.043 ± 0.000	0.055 ± 0.002	0.878 ± 0.004	0.055 ± 0.001	0.093 ± 0.002
CNN (color)	0.957 ± 0.001	0.043 ± 0.000	0.055 ± 0.001	0.879 ± 0.001	0.055 ± 0.001	0.092 ± 0.003
GNN (gcn)	0.984 ± 0.005	0.026 ± 0.004	0.032 ± 0.006	0.968 ± 0.010	0.028 ± 0.005	0.046 ± 0.010
GNN (gat)	0.986 ± 0.005	0.024 ± 0.004	0.029 ± 0.007	0.966 ± 0.009	0.029 ± 0.004	0.045 ± 0.006
GNN (gin)	**0.991** **±** **0.003**	**0.019** **±** **0.003**	**0.026** **±** **0.004**	**0.971** **±** **0.013**	**0.026** **±** **0.006**	**0.044** **±** **0.010**

The evaluation encompasses three distinct datasets, each corresponding to different inlet velocities: 0.01 m/s, 0.05 m/s, and a combined dataset that integrates both velocities. For both CNNs and GNNs, the performance metrics are summarized with their mean values and standard deviations, calculated across five different random initialization seeds. Bold values indicate the best results for each metric across different evaluation conditions.

### 3.1 Statistical ML results

The statistical methods exhibit high prediction accuracy when trained only on the 0.01 m/s inlet velocity data or when trained on the combined velocities, and among these methods, Elastic Net shows the strongest performance. In contrast, when trained using only the higher inlet velocity of 0.05 m/s, there is a marked drop in performance across all metrics. This performance gap suggests that statistical ML methods struggle to interpret the complex data resulting from increased turbulence and complexity at higher velocities. Comparing efficiency measures, the accuracy for interfacial area is almost always higher than for wetted area when the dataset includes the higher inlet velocity data.

This is likely because the interfacial area is more directly influenced by primary flow dynamics and solvent-gas interactions, whereas the wetted area involves more complex and variable factors, such as the distribution and retention of the solvent on the packing surfaces, which are highly sensitive to changes in turbulence and flow conditions, making it harder to model accurately. These effects become more pronounced at larger inlet velocities due to the increased turbulence and chaotic flow patterns, which exacerbate the variability in solvent distribution and retention, leading to greater prediction challenges (Prosperetti and Tryggvason, 2009; Panagakos and Shah, 2023).

Overall, the statistical ML methods can make accurate and rapid estimates of efficiency metrics, particularly with low inlet velocity data. However, they face challenges in predicting outcomes when higher inlet velocity data is involved, falling short compared to CNN- or GNN-based approaches. These advanced models are better equipped to handle complex spatial and relational data, leading to superior performance in predicting CO_2_-capture metrics, which we discuss in detail in the following sections.

### 3.2 CNN-based prediction results

As shown in Table 1, CNN-based methods demonstrate higher prediction accuracy with the higher inlet velocity model compared to the lower inlet velocity model, in contrast to the trend observed with statistical ML models. This improvement is attributed to the CNN's ability to effectively model data with greater complexity and turbulence. When trained and tested on the combined velocity dataset, the prediction accuracy improves over the single inlet velocity cases. This can be attributed to the larger volume of training data and the CNN's ability to learn from a more diverse set of flow conditions. Additionally, the difference in accuracy between the two efficiency measures is small, suggesting that CNNs effectively capture and interpret complex patterns in the data, whether they arise from turbulence or chaotic flow conditions.

Notably, CNNs demonstrate superior prediction accuracy for both efficiency metrics when trained and tested at the high inlet velocity. This highlights the importance of selecting appropriate data representations for our problem setting. Image-based representations, which incorporate packing geometries and even distinguish different physical components in the colored images, provide crucial spatial and contextual details that enhance the accuracy of predicting interfacial area and wetted area.

Furthermore, according to Table 1, the colored image model outperforms the grayscale image model in datasets that include the higher inlet velocity. This is also evident in Figures 2, 3, where the efficiency measure predictions are closer to the ground truth for the colored model. As data complexity increases, input representations with richer details (i.e., colored images) enable the model to extract more valuable information compared to the simplified representations (i.e., grayscale images). This underscores the benefits of using a three-channel input to differentiate various boundaries and structures within the column, highlighting the importance of choosing appropriate data representations.

In general, the performance of our CNN-based models highlights the advantages of using richer data representations for predicting CO_2_-capture efficiency metrics. Next, we discuss the GNN-based model, which further improves prediction accuracy by leveraging graph structures to represent the complex geometries and interactions within the CO_2_-capture columns.

### 3.3 GNN-based prediction results

Compared to the previous methods, the GNN-based methods achieve the highest prediction accuracy in most cases. Although there is a slight drop in accuracy for wetted area predictions across the three datasets, GNNs still maintain a high level of accuracy. Additionally, similar to statistical ML and CNNs, GNNs show a notable improvement in prediction accuracy when more data is available (i.e., combined inlet velocities) compared to using less data (i.e., single inlet velocity). This demonstrates that increasing both the quantity and variability of the data has a consistently positive effect across different learning-based models.

When comparing GNNs and CNNs, GNNs demonstrate superior overall performance, particularly excelling over CNNs when both velocity data sets are used for training. Such observation indicates that GNNs can better capture and model complex relationships, especially when more data is available. This underscores the robustness and adaptability of GNNs in handling diverse data complexities, making them a more reliable and favorable choice over CNNs for tasks requiring consistent accuracy across varying conditions. The superior generalizability of graph-based representations compared to image-based ones further enhances their effectiveness. Among three GNN models, GIN stands out with the best performance across most scenarios, while GCN demonstrates the most consistent results overall. GAT, though still effective, tends to have the lowest performance compared to GIN and GCN in most cases.

Overall, in addition to its high prediction accuracy, GNN-based methods offer greater flexibility in adapting to different packing geometries and scales due to its graph-based representation. Consequently, the GNN-based methods enable the evaluation of a broader range of designs and operational scenarios within a design optimization pipeline for CCS.

## 4 Discussion

In this work, we applied various ML models, including statistical ML methods, CNNs, and GNNs, to predict CO_2_-capture efficiency metrics. While statistical ML methods made fast and accurate estimates at lower inlet velocities, they struggled with higher velocities due to increased turbulence and complexity. Additionally, these models were limited to the 3-parameter packed column designs and scale of the training dataset columns. Conversely, CNN-based models, especially those using colored images, demonstrated high prediction accuracy, highlighting the importance of detailed data representations. However, the CNNs required a fixed image size as input, limiting their generalizability to different scales. In contrast, the GNN-based models consistently outperformed other methods due to their ability to capture complex relationships within graph-structured data. GNNs excel at learning structural and relational details, enabling them to adapt effectively to novel CCS column configurations, a crucial capability for real-world applications requiring modifications to column designs or operational conditions.

In summary, our results demonstrate that we can use ML models to estimate various CO_2_-capture efficiency measures without the need for additional CFD simulations. However, our approach still requires a large amount of data, and the CFD data that we summarized into efficiency measures is not fully utilized. An alternative approach would be to directly predict CFD simulations using ML-based methods and then compute the efficiency measures from those simulations. While this method would maximize the use of available data and potentially enhance prediction accuracy, it remains a challenging task due to the complexity of accurately modeling detailed CFD simulations. Future research may focus on overcoming these challenges to develop more efficient and effective prediction models.

## Data Availability

The raw data supporting the conclusions of this article will be made available by the authors, without undue reservation.
