# Supplementary material for: Comparative study of machine learning techniques for post-combustion carbon capture systems
Source: Front Artif Intell. 2024 Nov 14;7:1441934. doi: 10.3389/frai.2024.1441934 (PMC11603113; doi:10.3389/frai.2024.1441934)
Supplement: Supplementary file 1 [file Data_Sheet_1.pdf]

Supplementary Material

1 DATA PREPROCESSING

1.1 Train and Test Data Split

For each combination of inlet velocity and packing configuration, we generate a simulation in Ansys Fluent using a detailed two-phase reacting flow model (see Prosperetti and Tryggvason (2009); Brackbill et al. (1992); Panagakos and Shah (2023)) for further details). We use three types of data representations in our machine learning models: a 3-parameter representation $(\theta,\ H,\ d)$, an image-based representation of the CO_2-capture column, and a graph representation derived from the CFD mesh used to generate the simulations in Ansys Fluent. Specifically, we consider the following values: $\theta \in [30,\ 45,\ 65]$, $H \in [10,\ 13,\ 14.8]$, and $d \in [1.78,\ 2.68,\ 3.56]$. To assess the impact of turbulent data, we consider three dataset variants based on the inlet velocity: one using data with an inlet velocity of 0.01 m/s only, another with data at 0.05 m/s only, and a third combining data from both inlet velocities. We divide each dataset into training and test splits by applying Latin hypercube sampling (Loh, 1996) with the pyDOE package to ensure sufficient coverage of the 3-parameter space in the training set. In the combined velocity dataset, we include the inlet velocity value as an additional input feature for the model.

1.2 Data Preparation for CNN Models

To generate images based on packing geometry information, we create both gray-scale and color images, each serving different purposes. The color image, with a shape of 128 x 128 x 3 (right image in Figure S1(a)), uses three channels to distinctly represent various physical components of the column, with each component differentiated by unique colors: **column walls**, **packing**, **inlet**, **gas outlet**, and **pressure outlet**. This multi-channel approach provides a detailed visual distinction between different structures, aiding in the analysis of complex geometries. In contrast, the gray-scale image, with a shape of 128 x 128 x 1 (left image in Figure S1(a)), simplifies the representation by converting all features into shades of black and white. This approach eliminates distinctions between different types of boundaries and structures, streamlining the information into a single channel. We have experimented with different resolutions for these images and found that the 128 x 128 resolution offers the optimal balance. This resolution is large enough for the model to capture essential geometric features while being compact enough to reduce memory consumption. The 128 x 128 resolution provides sufficient detail for effective model learning without unnecessary computational overhead, making it the best choice for our purposes so far.

1.3 Data Preparation for GNN Models

We construct a graph by starting with the mesh representation of the packed columns used in the CFD simulations. To generate a triangular mesh for CFD from a given 2D geometry (see Figure S1(a)), the domain is first discretized into small, non-overlapping triangles. This process involves defining the boundary of the geometry and then filling the interior space with triangles, ensuring that the mesh accurately captures the geometry's shape and features. The quality of the mesh is crucial, as it influences the accuracy and stability of the CFD simulations. Key factors such as mesh density and triangle quality are adjusted based

on the complexity of the geometry and the desired level of detail. Once the triangular mesh is generated (see Figure S1(b)), it can be converted into a graph structure (see Figure S1(c)). In this conversion, each mesh node (or vertex) becomes a graph node, and edges are created between nodes corresponding to the sides of the triangles. This graph representation captures the connectivity of the mesh, enabling the use of graph-based algorithms to analyze or simulate physical phenomena. Nodes in the graph represent different locations in the column. To incorporate geometric information, we include each node's position and one-hot encoded type (represents different physical component) as node features, and we include relative positions and distances between nodes as edge features. This approach allows our GNN-based models to effectively capture the spatial relationships and geometric characteristics of the column's packing geometry, scale to arbitrary mesh sizes, and infer on novel column designs.

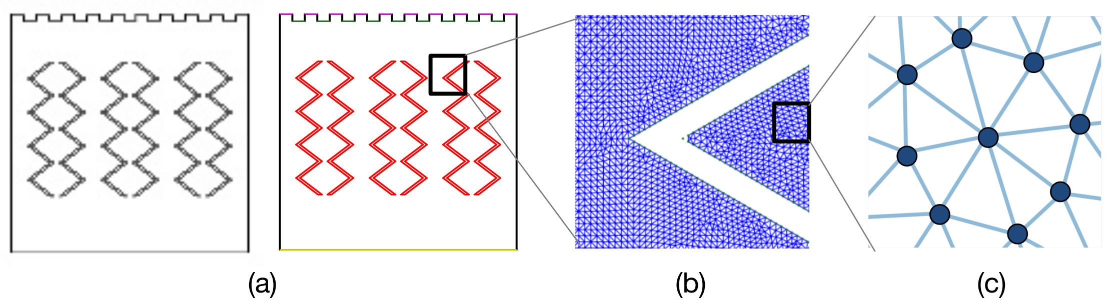

Figure S1. Illustration of the conversion process from CFD simulations to a graph structure. (a) Packing structure, (b) close-up view of the generated CFD mesh, and (c) close-up view of the triangular mesh structure and its conversion to a graph structure, where solid blue dots represent graph nodes and blue lines represent graph edges.

2 MODEL DETAILS

2.1 Convolutional Neural Networks (CNNs)

Figure S2(a) illustrates the detailed structure of CNN. Each convolutional block consists of a convolutional layer, a sigmoid activation function, and an average pooling operation. The convolutional layers use a kernel and a sigmoid activation function to map spatial inputs to 2D feature maps, typically increasing the number of channels. The first convolutional layer outputs 6 channels, and the second outputs 16. Each pooling operation reduces dimensionality by a factor of 4 through spatial downsampling. The convolutional block emits an output with shape given by (batch size, number of channel, height, width). To pass this output to the dense block, the four-dimensional input is flattened into a two-dimensional format suitable for fully-connected layers, where the first dimension indexes the minibatch, and the second represents each example as a flat vector. LeNet's dense block has three fully-connected layers with 120, 84, and 2 outputs, respectively. When handling data with varying inlet velocities, we include the inlet velocity as an input and use an additional linear layer to generate a 128-dimensional velocity embedding, which is then concatenated with the image embeddings just before the final output layer. To train the model, we use an Adam optimizer with a learning rate starting at 1e-4 over 3000 training steps. We use a batch size of 4 for data with consistent inlet velocity and 8 for data with different inlet velocity.

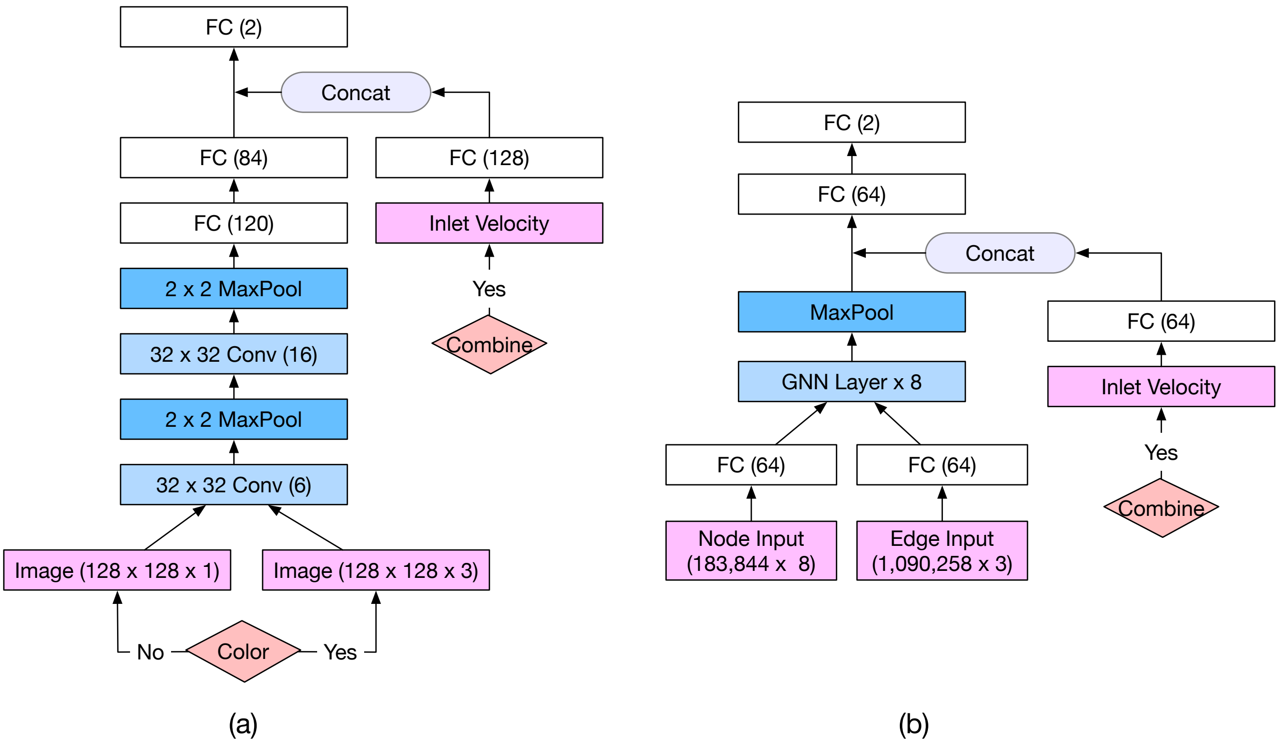

Figure S2. Illustration of (a) CNN structure and (b) GNN structure used in this paper.

2.2 Graph Neural Networks (GNNs)

On average, the graph of each configuration of θ, H, and d consists of 183,844 nodes and 1,090,258 edges. GNNs come in various architectures, each with unique approaches to aggregating and processing information across graph structures. In the paper we consider three commonly used models: Graph Convolutional Networks (GCN), Graph Attention Networks (GAT), and Graph Isomorphism Networks (GIN). **GCN** uses spectral convolution, aggregating neighbor features with equal weight, capturing structural information. **GAT** improves on this by applying attention mechanisms, assigning different importance to neighbors, which allows it to focus on key nodes. **GIN** maximizes expressive power, using a sum aggregation function to uniquely capture graph structures, making it as powerful as the Weisfeiler-Lehman test.

Figure S2(b) illustrates the detailed structure of GNN. To preprocess the mesh data for GNN, we encode node and edge features using a linear layer to obtain 64-dimensional latent feature embedding vectors, which are then passed through 8 GNN layers to perform message-passing and obtain node embeddings. We obtain the final graph embedding by applying max pooling to the node embeddings. Our experiments indicate that only considering nodes related to packing geometries during the pooling process yields better performance compared to pooling over all nodes. To incorporate inlet velocity as an input, we use an additional linear layer to generate a 64-dimensional velocity embedding. We then concatenate the graph and velocity embeddings and feed them through a 2-layer MLP to obtain an output prediction. To train the model, we use an Adam optimizer with a learning rate decayed from 1e-3 to 1e-5 over 1000 training steps and a batch size of 4.

2.3 Computation Time

Table S1 compares the computational performance of three methods: CFD, CNN, and GNN. For the CNN and GNN models, training and testing were conducted on a single NVIDIA V100 16 GB GPU. There is a significant difference in inference time between the CFD and machine learning models. CFD relies on iteratively solving complex fluid dynamics equations, which is computationally intensive and time-consuming. In contrast, CNN and GNN models, once trained, can make predictions almost instantaneously by leveraging pre-learned data patterns. The CNN model is approximately 2.44 million times faster than the CFD model, while the GNN model, despite having slightly longer inference times due to graph operations, is still 534,000 times faster than CFD. This dramatic reduction in computational time and resource demand underscores the potential of machine learning models in the post-combustion CO2 capture process.

Table S1. Computation Time Analysis For CFD, CNN and GNN.

	TRAINING TIME	INFERENCE TIME	COMPUTATION RESOURCES
CFD	N/A	157.4 h	160 CPUs
CNN	0.33 h	0.0067 s	1 GPU
GNN	0.18 h	1.06 s	1 GPU

3 RELATED WORKS

A post-combustion CO_2-capture process using chemical absorption involves an absorber and desorber in a closed loop. Flue gas with 10%–15% CO_2 enters the absorber from the bottom, while the absorbent flows downward, capturing CO_2. The CO_2-lean stream exits the absorber's top, while the CO_2-rich stream is sent to the desorber, where heat from a reboiler regenerates the absorbent. The regenerated absorbent returns to the absorber for further capture, and the pure CO_2 is collected after cooling. Trays or packing inside the columns ensure sufficient contact area (Wang et al., 2017).

3.1 Machine Learning for Carbon Capture System

A key challenge for large-scale CO_2-capture plants is the high energy demand, underscoring the need for a tool to model and optimize the process, reducing energy costs and maximizing capture rates. Traditional mechanistic models require extensive knowledge and are computationally intensive due to the process's nonlinear nature (Sipöcz et al., 2011; Li et al., 2017). In contrast, machine learning (ML) offers a data-driven approach that is less complex, computationally efficient, and can achieve accurate results using readily available process data. In Sipöcz et al. (2011), the authors employ a fully connected feed-forward network with one hidden layer to predict specific reboiler duty and solvent rich load, using the physical properties of the inlet flue gas as inputs. To predict the CO_2 production rate and capture level, (Li et al., 2015, 2017) employ bootstrap aggregated extreme learning machine (ELM) algorithms, while (Li et al., 2018) use a deep belief network (DBN) to extract a deep hierarchical representation of the training data. However, these studies only account for the influence of operating conditions (e.g., gas and solvent properties) on CO2 capture efficiency, overlooking the impact of packing geometries. Additionally, none of the works comprehensively compare the performance of different ML methods. In contrast, this work aims to evaluate both packing geometries and operating conditions across various ML models. The findings offer valuable

insights for selecting suitable ML algorithms and highlight the potential of these models in identifying optimal packing geometries and operating conditions.

3.2 GNNs for Dynamic System Prediction

The application of Graph Neural Networks (GNN) for dynamic system prediction is an emerging research area in scientific machine learning due to their versatility and effectiveness Belbute-Peres et al. (2020); Rubanova et al. (2021); Mrowca et al. (2018). Unlike image-based learning methods such as Convolutional Neural Networks (CNNs) Um et al. (2018); Ummenhofer et al. (2019), GNNs can directly handle unstructured simulation meshes, making them well-suited for simulating systems with complex domain boundaries while ensuring spatial invariance and locality Battaglia et al. (2018); Wu et al. (2020). The initial application of GNNs to physics-based simulations focused on deformable solids and fluids, with MeshGraphNets (MGN) being a pioneering work in this area Pfaff et al. (2020). MGN employs a message passing network to learn the dynamics of physical systems. Building on this foundation, various MGN variants have been proposed: integrating GNNs with Physics-Informed Neural Networks (PINNs) **?**, enabling long-term predictions by combining GraphAutoEncoder (GAE) and Transformer models Han et al. (2022), directly predicting steady states through multi-layer readouts Harsch and Riedelbauch (2021), and accelerating fine-level simulations by using up-sampled coarse results inferred by GNNs Belbute-Peres et al. (2020).

DATA AVAILABILITY STATEMENT

Code and datasets for this study are available upon request.

REFERENCES

Battaglia, P. W., Hamrick, J. B., Bapst, V., Sanchez-Gonzalez, A., Zambaldi, V., Malinowski, M., et al. (2018). Relational inductive biases, deep learning, and graph networks. arxiv 2018. *arXiv preprint arXiv:1806.01261*

Belbute-Peres, F. D. A., Economon, T., and Kolter, Z. (2020). Combining differentiable pde solvers and graph neural networks for fluid flow prediction. In *international conference on machine learning* (PMLR), 2402–2411

Brackbill, J. U., Kothe, D. B., and Zemach, C. (1992). A continuum method for modeling surface tension. *Journal of computational physics* 100, 335–354

Han, X., Gao, H., Pfaff, T., Wang, J.-X., and Liu, L.-P. (2022). Predicting physics in mesh-reduced space with temporal attention. *arXiv preprint arXiv:2201.09113*

Harsch, L. and Riedelbauch, S. (2021). Direct prediction of steady-state flow fields in meshed domain with graph networks. *arXiv preprint arXiv:2105.02575*

Li, F., Zhang, J., Oko, E., and Wang, M. (2015). Modelling of a post-combustion co2 capture process using neural networks. *Fuel* 151, 156–163

Li, F., Zhang, J., Oko, E., and Wang, M. (2017). Modelling of a post-combustion co 2 capture process using extreme learning machine. *International Journal of Coal Science & Technology* 4, 33–40

Li, F., Zhang, J., Shang, C., Huang, D., Oko, E., and Wang, M. (2018). Modelling of a post-combustion co2 capture process using deep belief network. *Applied Thermal Engineering* 130, 997–1003

Loh, W.-L. (1996). On latin hypercube sampling. *The annals of statistics* 24, 2058–2080

Mrowca, D., Zhuang, C., Wang, E., Haber, N., Fei-Fei, L. F., Tenenbaum, J., et al. (2018). Flexible neural representation for physics prediction. *Advances in neural information processing systems* 31

Panagakos, G. and Shah, Y. G. (2023). *A Computational Investigation of the Effect of Packing Structural Features on the Performance of Carbon Capture for Solvent-Based Post-Combustion Applications*. Tech. rep., National Energy Technology Laboratory (NETL), Pittsburgh, PA

Pfaff, T., Fortunato, M., Sanchez-Gonzalez, A., and Battaglia, P. W. (2020). Learning mesh-based simulation with graph networks. *arXiv preprint arXiv:2010.03409*

Prosperetti, A. and Tryggvason, G. (2009). *Computational methods for multiphase flow* (Cambridge university press)

Rubanova, Y., Sanchez-Gonzalez, A., Pfaff, T., and Battaglia, P. (2021). Constraint-based graph network simulator. *arXiv preprint arXiv:2112.09161*

Sipöcz, N., Tobiesen, F. A., and Assadi, M. (2011). The use of artificial neural network models for co2 capture plants. *Applied Energy* 88, 2368–2376

Um, K., Hu, X., and Thuerey, N. (2018). Liquid splash modeling with neural networks. In *Computer Graphics Forum* (Wiley Online Library), vol. 37, 171–182

Ummenhofer, B., Prantl, L., Thuerey, N., and Koltun, V. (2019). Lagrangian fluid simulation with continuous convolutions. In *International Conference on Learning Representations*

Wang, Y., Zhao, L., Otto, A., Robinius, M., and Stolten, D. (2017). A review of post-combustion co2 capture technologies from coal-fired power plants. *Energy Procedia* 114, 650–665

Wu, Z., Pan, S., Chen, F., Long, G., Zhang, C., and Philip, S. Y. (2020). A comprehensive survey on graph neural networks. *IEEE transactions on neural networks and learning systems* 32, 4–24
